# The effect of tracer contact on return to care among adult, “lost to follow‐up” patients living with HIV in Zambia: an instrumental variable analysis

**DOI:** 10.1002/jia2.25853

**Published:** 2021-12-18

**Authors:** Laura K. Beres, Aaloke Mody, Kombatende Sikombe, Lauren Hersch Nicholas, Sheree Schwartz, Ingrid Eshun‐Wilson, Paul Somwe, Sandra Simbeza, Jake M. Pry, Paul Kaumba, John McGready, Charles B. Holmes, Carolyn Bolton‐Moore, Izukanji Sikazwe, Julie A. Denison, Elvin H. Geng

**Affiliations:** ^1^ Department of International Health Johns Hopkins Bloomberg School of Public Health Baltimore Maryland USA; ^2^ Department of Medicine Washington University School of Medicine St. Louis Missouri USA; ^3^ Centre for Infectious Disease Research in Zambia Lusaka Zambia; ^4^ Department of Health Policy and Management Johns Hopkins Bloomberg School of Public Health Baltimore Maryland USA; ^5^ Department of Epidemiology Johns Hopkins Bloomberg School of Public Health Baltimore Maryland USA; ^6^ Department of Biostatistics Johns Hopkins Bloomberg School of Public Health Baltimore Maryland USA; ^7^ Center for Innovation in Global Health Georgetown University Washington DC USA; ^8^ Division of Infectious Disease Johns Hopkins University School of Medicine Baltimore MD USA; ^9^ Department of Medicine University of Alabama at Birmingham Birmingham Alabama USA

**Keywords:** causality, HIV, instrumental variable, lost to follow‐up, retention in care, tracing

## Abstract

**Introduction:**

Tracing patients lost to follow‐up (LTFU) from HIV care is widely practiced, yet we have little knowledge of its causal effect on care engagement. In a prospective, Zambian cohort, we examined the effect of tracing on return to care within 2 years of LTFU.

**Methods:**

We traced a stratified, random sample of LTFU patients who had received HIV care between August 2013 and July 2015. LTFU was defined as a gap of >90 days from last scheduled appointment in the routine electronic medical record. Extracting 2 years of follow‐up visit data through 2017, we identified patients who returned. Using random selection for tracing as an instrumental variable (IV), we used conditional two‐stage least squares regression to estimate the local average treatment effect of tracer contact on return. We examined the observational association between tracer contact and return among patient sub‐groups self‐confirmed as disengaged from care.

**Results:**

Of the 24,164 LTFU patients enumerated, 4380 were randomly selected for tracing and 1158 were contacted by a tracer within a median of 14.8 months post‐loss. IV analysis found that patients contacted by a tracer because they were randomized to tracing were no more likely to return than those not contacted (adjusted risk difference [aRD]: 3%, 95% CI: –2%, 8%, *p* = 0.23). Observational data showed that among contacted, disengaged patients, the rate of return was higher in the week following tracer contact (IR 5.74, 95% CI: 3.78–8.71) than in the 2 weeks to 1‐month post‐contact (IR 2.28, 95% CI: 1.40–3.72). There was a greater effect of tracing among patients lost for >6 months compared to those contacted within 3 months of loss.

**Conclusions:**

Overall, tracer contact did not causally increase LTFU patient return to HIV care, demonstrating the limited impact of tracing in this program, where contact occurred months after patients were LTFU. However, observational data suggest that tracing may speed return among some LTFU patients genuinely out‐of‐care. Further studies may improve tracing effectiveness by examining the mechanisms underlying the impact of tracing on return to care, the effect of tracing at different times‐since‐loss and using more accurate identification of patients who are truly disengaged to target tracing.

## 1 INTRODUCTION

Consistent engagement in HIV care is critical for patient health [1, 2, 3, 4, 5] and achieving global epidemic control targets [6]. Retention is challenging, however, with approximately one‐third of patients on antiretroviral therapy (ART) [7] in sub‐Saharan Africa considered lost to follow‐up (LTFU). While some patients return to care, many do not. Observational estimates of re‐engagement in care in Africa and the United States range from one‐quarter to three‐quarters [8, 9, 10, 11, 12, 13]. As HIV programmes transition from an emergency‐focused response to a long‐term maintenance service delivery model, they must allocate limited resources to interventions that support sustained patient engagement [14].

Tracing patients LTFU from HIV care is widely practiced, yet we have little knowledge of its causal effect on care engagement. Tracing patients identified as having missed an HIV visit using phone calls or home visits is the standard of care in many countries, and the U.S. President's Emergency Plan for AIDS Relief requires it [15]. Although useful in determining patient outcomes among those LTFU [16, 17, 18, 19, 20, 21], there is limited evidence of its effectiveness re‐engaging out‐of‐care patients. Several studies [22, 23, 24, 25] of variable quality [26] have assessed the impact of tracing on return to care, producing effect estimates ranging from null to a significant improvement in re‐engagement. Rigorous causal evidence is needed to guide the use of tracing.

Using an instrumental variable (IV) approach, we estimated the causal effect of tracing on return to care within 2 years of patient loss in a prospective cohort of Zambian patients. This analysis informs interventions to support HIV care engagement continuity.

## 2 METHODS

Our study leveraged the “Better Information for Health in Zambia” (BetterInfo) study, which generated representative estimates of HIV programme outcomes by tracing a large, random sample of LTFU patients to ascertain their outcomes (i.e. undocumented in‐care, out‐of‐care and deceased) [17, 18]. Our study exploited the fact that BetterInfo patients were randomly selected for tracing to use random selection for tracing as an IV. BetterInfo was conducted in 71 public health facilities supported by the Zambian Ministry of Health and Centre for Infectious Disease Research in Zambia across four provinces: Eastern, Lusaka, Southern and Western.

### 2.1 Study population

Drawing from BetterInfo, our study population included patients who had at least one HIV care visit between 1st August 2013 and 31st July 2015 at a study facility and were then LTFU, defined as a care gap of >90 days according to the electronic medical record (EMR) and subsequent unknown care status. We conducted the study during a period with free HIV‐related care for all patients, ART eligibility CD4 <500 and stable ART patients receiving 90–180 days ART supply [27].

### 2.2 Procedures and measurements

The BetterInfo study randomly sampled patients for tracing by first enumerating the 2013–2015 patient population across 71 facilities. Thirty‐one facilities, where tracing would occur, were selected using probability proportional to size sampling from strata defined by facility type (hospital, urban or rural health centre) and province. LTFU patients were then selected through simple random sampling at facility level [17]; selected patients were traced in a random order per facility.

Tracing occurred from 23rd September 2015 through 17th July 2016 by study‐trained peer educators knowledgeable about the community served by the facility from which they conducted tracing (tracers). Tracing included: (1) reviewing the patient's paper medical file for confirmed death or recent facility visit not documented in the EMR. If found, the patient outcome was recorded and tracing ended. (2) If an outcome was not established, tracers attempted to contact the patient using phone calls and in‐person visits guided by contact information in the patient's medical file [17]. Tracers made at least three in‐person attempts to contact a patient over several months until an updated status was ascertained by the tracer and recorded in the study survey form, or the tracing period ended.

Upon contact, tracers verbally encouraged disengaged patients to return to care. Although not systematically offered, tracers sometimes accompanied patients to the facility or met them there for their return visit. Tracers made no further follow‐up after one‐time, successful in‐person contact. Tracer contact with the patient was documented, including the contact date and means of communication (phone or in‐person), using the study tracing form, and stored in an electronic study database. LTFU patients not sampled for tracing were not precluded from non‐study, facility‐led follow‐up; however, little non‐study follow‐up occurred during the study period.

**Figure 1:**
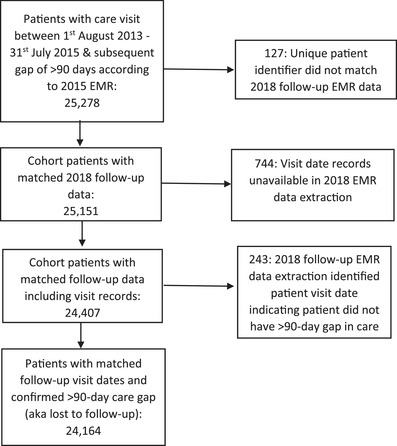
Analysis population.

Our study outcome, *return to care*, was measured using EMR visit data. In 2018, we extracted 2 years of follow‐up patient visit data (through 2017) from all LTFU patients in the 2015 cohort. We linked follow‐up visit records through unique EMR patient identification numbers (Figure 1) at the 71 sites. Thus, our study population included patients who, since their last EMR‐documented facility visit in 2013–2015, were deceased, were alive and in care, alive but not seeking care or deceased. The date of patient loss from care and demographic, social and clinical characteristics at the time of loss were extracted from the EMR. Missing data were imputed using chained equations [28] to create a single imputed dataset.

### 2.3 Analysis

We defined patients as *returned to care* (the outcome variable) if they had a visit date in the EMR within 2 years following their loss date. The return date was the first facility visit date post‐loss. We considered patients to have received the treatment if they were contacted over the phone or in person by a study tracer before their return visit or within 2 years after loss.

#### 2.3.1 Intention‐to‐treat analysis

To perform an intention‐to‐treat (ITT) analysis, we used the Kaplan–Meier method to estimate the cumulative incidence of return within 2 years according to random treatment assignment (randomized to study tracing or not) and a log‐rank test to compute the associated *p*‐value. The time origin was the date of patient loss (missed, scheduled appointment date+90 days or last clinic visit+180 days if scheduled appointment undocumented in EMR). The time scale was days‐since‐loss. The event was patient return or censor at 2 years post‐loss.

#### 2.3.2 Instrumental variable analysis

To estimate the causal effect of tracing on return, we leveraged the experiment inherent in the BetterInfo study design, which randomly assigned patients to tracing. The association between successful tracer–patient contact (i.e. the treatment) and a patient returning to care within 2 years post‐loss (i.e. the outcome) was likely confounded by numerous, unmeasured common causes of a tracer's ability to make contact and the patient's return behaviour. In short, the same things that enable a tracer to successfully contact a patient likely also help the patient to return.

Using *randomization to tracing* as an IV can yield an unbiased estimate, even in the presence of unmeasured confounding, provided that it meets several key assumptions: (1) the IV (randomization to tracing) is associated with the treatment (successful tracer–patient contact), (2) there are no common causes between being randomized to tracing and the outcome, (3) the effect of the randomization to tracing on patient return is fully mediated through the tracer–patient contact (the treatment), (4) randomization to tracer contact will only increase, not decrease, a patient's likelihood of tracer contact (monotonicity) and (5) one patient's randomization to tracing does not affect another patient's return to care (stable unit treatment value assumption) [29, 30, 31]. Under these assumptions, the IV analysis estimates the causal effect of tracer–patient contact on return to care among patients who would not have been contacted by a tracer had they not been randomized to tracing (the local average treatment effect [LATE]) [29]. Assumptions 1, 3, 4 and 5 are supported by the study's design or theoretically justified [29] (Table 1). We empirically examined assumptions 1 and 2.

**Table 1:** Assumptions necessary for a valid instrumental variable (IV) analysis

Assumption	Theoretical justification
1. The instrumental variable (randomization to tracing) is associated with the treatment (successful tracer–patient contact)	Based on the study design, only patients randomized to tracing could be contacted by a study tracer. Not being randomly selected for tracing identifies those who did not have tracer contact. (Examined empirically in the first stage of two‐stage least squares regression.)
2. There are no unmeasured common causes between randomization to tracing and the outcome	Randomized assignment to tracing should balance patient characteristics between the two arms, eliminating unmeasured confounding. (Examined empirically assessing balance in measured potential confounders.)
3. The effect of the randomization to tracing on patient return is fully mediated through the tracer–patient contact	The instrumental variable, randomization to tracing, was only used to determine study tracing interactions. It did not affect any other treatments that a patient received.
4. Monotonicity (i.e. no “defiers”): Randomization to tracing only increased the likelihood of patient–tracer contact [32]	Only participants randomized to tracing could have received tracer–patient contact provided by the study. No one was prevented from being traced because they were randomized to tracing.
5. Stable unit treatment value assumption: one patient's randomization to tracing does not affect another patient's outcome	Patients were unaware of their randomization status. Randomization was only linked to study tracing.

To evaluate assumption 1 and estimate the LATE risk difference (RD), we used two‐stage least squares regression (2SLSR) using ivreg2 in Stata [33]. To improve the robustness of the analysis, given the binary outcome, we also estimated the marginal RD with binary probit regression [34] using the biprobit Stata command. We used descriptive statistics to examine covariate balance between the two treatment arms (examining assumption 2 to the extent possible) and to estimate the proportion of patients assigned to tracing who were successfully contacted by a tracer (“compliers”). The instrument was randomly assigned, but we conditioned on covariates due to outcomes from the stratified sampling schema. These covariates showed an imbalance between the two treatment assignments, including ART initiation status, time from enrolment to loss, prior gaps in care and facility type (Table 2). For model inclusion, the relationship between covariates and the outcome was assessed using descriptive statistics for categorical and LOWESS plots for continuous covariates. Based on hypotheses of causal relationships and empirical review of patient characteristics, we believe that it is plausible to assume no other unmeasured confounding.

**Table 2:** Patient characteristics by randomization to tracing (instrumental variable)

	Not randomized to tracing^ŧ^		Randomized to tracing^ŧ^		Total	
	*n* = 19,784		*n* = 4380		24,164	
	*n*	%	*n*	%	*n*	*p*‐value*
*Characteristics*						
Sex					24,164	0.94
Female	11,935	60.3	2,645	60.4	14,580	
Male	7849	39.7	1735	39.6	9584	
Age at loss (years)					24,102	0.16
18–24	2069	10.5	473	10.9	2542	
25–34	7425	37.6	1588	36.4	9013	
35–44	6686	33.9	1456	33.4	8142	
45+	3563	18.0	842	19.3	4405	
Ever initiated antiretroviral therapy					24,164	<0.01
No	5819	29.4	1521	34.7	7340	
Yes	13,965	70.6	2859	65.3	16,824	
Weeks from time of enrolment to loss (mean, standard deviation)	18.4	18.5	16.8	17.1	24,164	<0.01^∧^
Weeks from date of loss‐to‐follow‐up to date of randomization (mean, standard deviation)	42.5	25.7	42.8	25.7	24,164	0.45^∧^
Number of prior gaps in care					24,164	<0.01
0 gaps	10,343	52.3	2478	56.6	12,821	
1 gap	5065	25.6	1057	24.1	6122	
2–3 gaps	3797	19.2	714	16.3	4511	
4 gaps or more	579	2.9	131	3.0	710	
Facility type					24,164	<0.01
Rural health centre	1522	7.7	996	22.7	2518	
Urban health centre	13,902	70.3	2283	52.1	16,185	
Hospital	4360	22.0	1101	25.1	5461	
Marital status					19,703	0.55
Single, never married	2161	13.4	509	14.2		
Married	10,225	63.5	2237	62.4		
Divorced	2316	14.4	528	14.7		
Widowed	1413	8.8	314	8.8		
Education					19,073	<0.01
No formal education	918	5.9	338	9.9	1256	
Lower‐mid basic	5580	35.6	1259	36.9	6839	
Upper basic – secondary	8475	54.1	1662	48.6	10,137	
Tertiary	683	4.4	158	4.6	841	
Last CD4 count (cells/μmol) before loss					20,575	0.04
< = 100	1976	11.5	408	11.9	2384	
101–350	6255	36.5	1246	36.2	7501	
351–500	3717	21.7	681	19.8	4398	
>500	5189	30.3	1103	32.1	6292	
*Treatment*						
Patient contacted by tracer before return or 2 years follow‐up					24,164	<0.01
No	19,784	100.0	3222	73.6	23,006	
Yes	0	0.0	1158	26.4	1158	
*Outcome*						
Return visit within 2 years of loss					24,164	0.04
No	14,811	74.9	3212	73.3	18,023	
Yes	4973	25.1	1168	26.7	6141	

* chi‐square test.

^∧^ *t*‐test.

^ŧ^Percentages may not add to 100 due to rounding.

#### 2.3.3 Instrumental variable analysis sensitivity analyses

To examine potential differential effects by strata, we conducted stratified analyses by (1) ART initiation status at loss, (2) health facility and (3) time from loss to randomization assignment.

There was a gap of approximately 6 weeks between when patients could be identified as LTFU (31st July 2015) and randomization assignment (15th September 2015), after which treatment exposure was possible. We conducted a sensitivity analysis estimating the LATE on patients who remained LTFU at randomization (no EMR‐documented return visit between lost date and date of randomization in follow‐up data). Exposure was tracer contact with the patient before return or within 2 years after randomization, and an outcome was a facility visit within 2 years after randomization. We controlled for time from loss to randomization, in addition to other covariates.

#### 2.3.4 Incidence rate of return relative to tracing exposure in patient sub‐groups

The causal analysis limits assessment of patient sub‐groups, such as LTFU patients known to be out‐of‐care, which may be of interest in programmatic intervention implementation. We estimated the incidence rate (IR) of return relative to different tracing experiences among patient sub‐groups; this allowed us to assess the observational association between tracer–patient contact and return to care. We estimated the IR: (1) throughout the 2 years of follow‐up among all patients, randomized to tracing or not, (2) in the 2 weeks before and 2 weeks after tracer contact among patients who had contact with a tracer (in‐person, on the phone or through a relation and were confirmed to be alive), (3) at varying time points post‐tracer‐contact among patients who had phone or in‐person contact with a tracer and who were confirmed to be alive and disengaged from care at the time of tracer contact. The time origin, scale, event and censor were the same as in the ITT analysis.

Analyses were conducted using Stata 15.1 IC (StataCorp, 2018).

### 2.4 Ethical review

This study was approved by the University of Zambia Research Ethics Committee, the Zambian Ministry of Health and the University of Alabama at Birmingham Institutional Review Board, which determined that programmatic EMR data analysis did not require additional consent. Voluntary, written informed consent was obtained following in‐person patient–tracer contact.

## 3 RESULTS

### 3.1 Cohort description

We included 24,164 participants in the analysis (Figure 1). The majority of the cohort was female (*n* = 14,580, 60.3%), with a median age at LTFU of 35.5 years (IQR: 29.7–42.3), median CD4 at last visit of 363 cells/μmol (IQR: 200–550), mean 43 weeks from loss to randomization assignment (SD: 26, min: 7, max: 98 weeks) and 69.6% (*n* = 16,824) ART‐initiated (Table 3). Among the 4380 patients randomized to tracing, 13% died, 23% were alive and in‐care at their original facility, 14% were alive and in‐care at a different facility, 13% were alive and disengaged from care, 8% were alive with unknown care status and in 30% tracing was unsuccessful. Of the 3034 patients eligible for phone or household tracing after paper health record review, 1158 had tracer contact before return or 2 years of follow‐up (the treatment), which is 26.4% of patients randomized to tracing (proportion of compliers) (Figure S1). Most tracer–patient contacts, 95%, were in‐person, with 5% over the phone. Among those successfully contacted, median time from patient loss to tracer contact was 14.8 months (IQR: 10.8–18.8).

**Table 3:** Patient characteristics at time of loss to follow‐up by tracer contact

	No tracer contact before return or 2 years follow‐up^ŧ^		Tracer contact before return or 2 years follow‐up^ŧ^		Total	
	*n* = 23,006		*n* = 1158		24,164	
	*n*	%	*n*	%	*n*	*p*‐value*
*Characteristics*						
Sex					24,164	0.11
Female	13,855	60.2	725	62.6	14,580	
Male	9151	39.8	433	37.4	9584	
Age at loss (years)					24,102	<0.01
18–24	2388	10.4	154	13.3	2542	
25–34	8553	37.3	460	39.8	9013	
35–44	7761	33.8	381	33.0	8142	
45+	4245	18.5	160	13.9	4405	
Ever initiated antiretroviral therapy					24,164	<0.01
No	6795	29.5	545	47.1	7340	
Yes	16,211	70.5	613	52.9	16,824	
Weeks from time of enrolment to loss (mean, standard deviation)	18.3	18.3	13.8	15.6	24,164	<0.01^∧^
Weeks from date of loss to follow‐up to date of randomization (mean, standard deviation)	42.7	25.9	40.5	21.2	24,164	0.01^∧^
Number of prior gaps in care					24,164	<0.01
0 gaps	12,108	52.6	713	61.6	12,821	
1 gap	5871	25.5	251	21.7	6122	
2–3 gaps	4340	18.9	171	14.8	4511	
4 gaps or more	687	3.0	23	2.0	210	
Facility type					24,164	<0.01
Rural health centre	2257	9.8	261	22.5	2518	
Urban health centre	15,584	67.7	601	51.9	16,185	
Hospital	5165	22.5	296	25.6	5461	
Marital status					19,703	0.21
Single, never married	2524	13.5	146	15.0	2670	
Married	11,834	63.2	628	64.3	12,462	
Divorced	2719	14.5	125	12.8	2844	
Widowed	1650	8.8	77	7.9	1727	
Education					19,073	0.12
No formal education	1183	6.5	73	8.0	1256	
Lower‐mid basic	6532	36.0	307	33.6	6839	
Upper basic – secondary	9652	53.2	485	53.1	10,137	
Tertiary	793	4.4	48	5.3	841	
Last CD4 count (cells/μmol) before loss					20,575	<0.01
< = 100	2318	11.8	66	7.4	2384	
101–350	7193	36.5	308	34.6	7501	
351–500	4201	21.3	197	22.1	4398	
>500	5973	30.3	319	35.8	6292	

* chi‐square test.

^∧^ *t*‐test.

^ŧ^Percentages may not add to 100 due to rounding.

### 3.2 Intention‐to‐treat analysis

**Figure 2:**
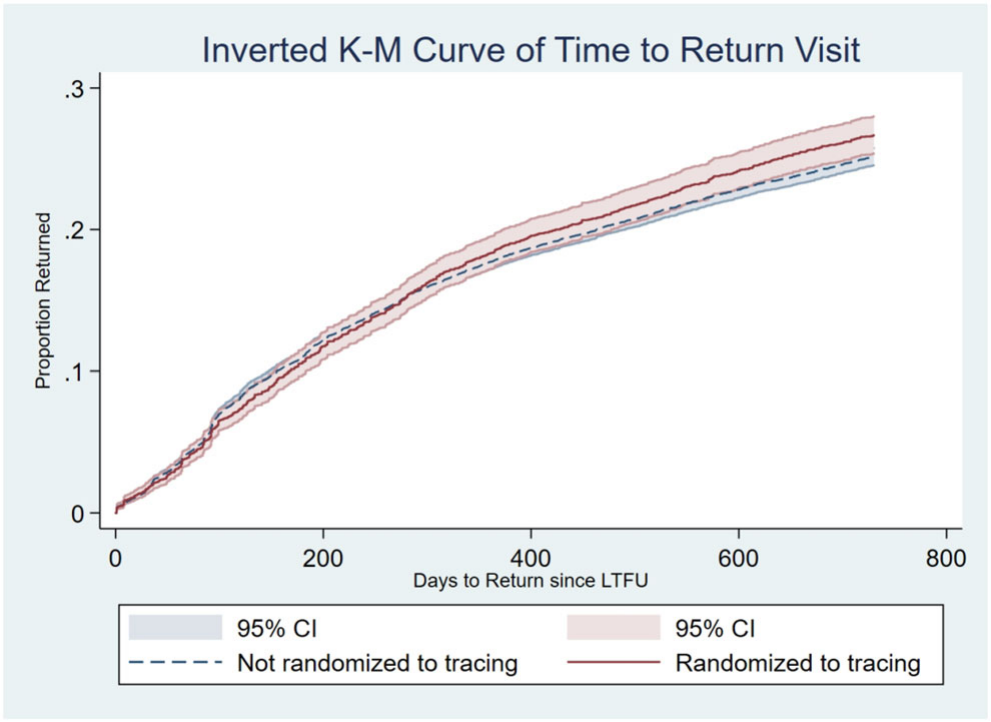
Kaplan–Meier failure estimate of return to care by 2 years post‐loss stratified by randomization to tracing.

Patients randomized to tracing were not significantly more likely to return to care, with a 1.6% absolute difference in the failure function (not randomized to tracing: 25.1%, 95% CI: 24.5–25.8%; randomized to tracing: 26.7%, 95% CI: 25.4–28.0%, *p*‐value 0.06) (Figure 2). The crude estimated hazard ratio of return for randomization to tracing is 1.06 (95% CI: 1.00–1.13, *p*‐value 0.06).

### 3.3 Instrumental variable analysis

Regressing the treatment on the IV in the first stage of the adjusted 2SLSR supports assumption #1, showing that randomization to tracing is strongly associated with tracer–patient contact, first‐stage partial *r* ^2^ = 0.22, *f*‐statistic = 6805.30, *p*<0.01, aRD 26.4%, 95% CI: 25.7–27.0%) [29].

Patients who were contacted by a tracer because they were randomized to tracing were not significantly more likely to return than those not contacted (aRD: 3%, 95% CI: –2%, 8%, *p* = 0.23) (Table 4). The adjusted effect estimate was similar using bivariate probit regression (aRD: 5%, 95% CI: –2%, 11%, *p* = 0.16) (Table 4). The 2SLSR and bivariate probit models (error term rho chi^2^ = 16.2, *p* <0.01) suggest that selection bias is a concern in descriptive regressions, demonstrating the importance of controlling for unmeasured confounding (Table 4; effect estimates for all included covariates: see Table S4).

**Table 4:** Instrumental variable estimate: treatment effect of tracer contact on return to care among patients LTFU

	Two‐stage least squares regression, adjusted*, [Link]					Binary probit regression, adjusted*, ^#^		
	Risk difference	95% CI		*p*‐value	Risk difference	95% CI		*p*‐value
Patient contacted by tracer before return or 2 years follow‐up versus not	0.03	–0.02	0.08	0.23	0.05	–0.02	0.11	0.16

* Adjusted for sex, age at LTFU, ART initiation status, time since HIV care enrolment, prior care gaps, facility type, highest education attained and CD4 at last visit before loss.

First stage: adjusted RD 0.26 (95% CI: 0.26, 0.27, *p* = <0.01), partial *r* ^2^ = 0.22, *F* = 6805.30.

^∧^Ordinary least squares regression (OLS), crude: –0.08 (95% CI: –0.10, –0.05, *p* = <0.01); OLS, adjusted: –0.07 (95% CI: –0.09, –0.04, *p* = <0.01).

^#^ Probit regression, crude: –0.27 (95% CI: –0.35, –0.18, *p* = <0.01); probit regression, adjusted: –0.23 (95% CI: –0.32, –0.14, *p* = <0.01).

The findings were robust and maintained in sensitivity analyses (Tables S1–S3). While confidence intervals were wide, facility‐level stratified analyses demonstrated effect heterogeneity (Figure S2). In analyses stratified by time‐since‐loss, there was a greater effect of tracer–patient contact among patients lost for >6 months than those contacted within 3 months of loss (Figure S3).

### 3.4 Incidence rate of return

Among patients who spoke with a tracer and were confirmed as disengaged from care at the time of tracer contact, the IR of return to care was higher in the week following tracer contact (IR 5.74, 95% CI: 3.78–8.71) than in the 2 weeks to 1 month following tracer contact (IR 2.28, 95% CI: 1.40–3.72) and in subsequent time periods (Table 5). Overall, 117 of the disengaged patients with tracer contact returned to care within 2 years of loss, with 18.8% returning the first week post‐tracer contact, 28% within the 2 weeks post‐tracer contact and 42% within a month of tracer contact. Among patients of any care status (in‐care, disengaged and unknown status) confirmed to be alive through tracer contact, the rate of return was higher in the 2 weeks after tracer contact (IR 3.03, 95% CI: 2.34–3.94) than in the 2 weeks before tracer contact (IR 0.59, 95% CI: 0.32–1.10) (Table 5).

**Table 5:** Incidence rate of return among patient sub‐groups at varying periods relative to tracing

Patient sub‐group	Period	*n* ^a^	Incidence rate per 1000 py	95% CI
All patients in study not randomized to tracing	2 years of study follow‐up	19,784	0.41	0.40–0.42
All patients in study randomized to tracing	2 years of study follow‐up	4380	0.44	0.41–0.46
Phone or in‐person contact between tracer and patient, or tracer and person known to patient, patient confirmed alive (includes in‐care, disengaged and unknown care status)	2 weeks before tracer interaction	1310	0.59	0.32–1.10
	2 weeks after tracer interaction	1283	3.03	2.34–3.94
Phone or in‐person contact between tracer and patient, patient confirmed alive and disengaged from care at time of tracer contact	Up to 1 week after tracer contact	496	5.74	3.78–8.71
	1–2 weeks after tracer contact	468	3.42	1.90–6.18
	2 weeks–1 month after tracer contact	450	2.28	1.40–3.72
	1–2 months after tracer contact	424	1.56	1.00–2.45
	2–6 months after tracer contact	387	0.74	0.51–1.06

Abbreviation: py, patient years.

^a^ Number of patients who did not exit (return or reach 2 years of follow‐up post‐loss) by period of interest.

## 4 DISCUSSION

Tracer contact did not significantly increase return to HIV care within 2 years of loss in a prospective cohort of patients from 31 facilities across four provinces in Zambia. Using an IV approach, the causal effect of tracer–patient contact on return to care among LTFU patients was a non‐significant increase of 3% (95% CI:–2%, 8%). Tracing may be more effective among patients remaining lost for >6 months–1 year (13%, 95% CI: 0.06–0.20) and >1 year (7%, 95% CI: 0.01–0.13) compared to <3 months (–21%, 95% CI: –0.42, –0.01). Observational data suggest that tracer interaction speeds return among truly disengaged patients; the estimated rate of return was significantly higher in the week immediately following tracer contact (IR 5.74, 95% CI: 3.78–8.71) compared to return during the 2 weeks to 1‐month post‐contact (IR 2.28, 95% CI: 1.40–3.72), and any subsequent time.

While tracing has increased engagement in other disease areas, such as cervical cancer [35] and tuberculosis [36], it has yielded small effect sizes that vary over time. Programmatic data have shown patients living with HIV to be less responsive to tracing than patients with other chronic diseases [22]. Our findings expand the extant literature on the effect of tracing on re‐engagement in HIV care [20, 37, 38], suggesting that broadly applying tracing to all LTFU patients, as defined by a > 90‐day gap in the EMR, may have limited effect.

As with any IV, we must consider the effect estimate's external validity [32]. The causal effect estimate applies to patients who would not have had tracer contact had they not been randomized to tracing (i.e. the LATE). At the time of our study, non‐study tracing efforts were limited. Additionally, we used EMR data and a standard definition of LTFU (>90 days from last missed appointment) to identify patients for tracing, a common process in non‐study settings. Our findings are thus likely applicable in regional programmatic settings to clinic‐based service delivery models.

Compared with the study period, current HIV programmatic tracing is more active, targeted at re‐engagement and attempted as soon as possible after missed appointments [15]. In our study, tracing occurred a median of 14.8 months after LTFU. Other studies have shown benefits of early tracing [39], which our study treatment may not capture. Further, tracing timing may have a differential impact depending on barriers to care [9, 11, 40]. Our findings suggest that, while rapid return is preferable, the use of tracing as a re‐engagement tool may be best applied after a certain threshold, such for those as remaining lost for 6 months.

Qualitative studies and conceptual work demonstrate that re‐engagement is complex [40, 41, 42, 43, 44]. A single intervention, such as tracing, may contribute a small effect when applied across a broad HIV service programme. Our observational data on truly disengaged patients showed an increased rate of return soon after a tracing interaction. The disengaged patients represent only those tracers were able to reach, likely giving a biased effect estimate. However, patients traceable in the study were probably similar to those that a programmatic tracing effort could successfully contact in real‐world implementation.

The study's tracing efforts were extensive, including multiple attempts at patient contact and interpersonal communication supportive of return [17]. They were, at a minimum, consistent with standard health facility verbal encouragement to return. Studies from higher resourced settings have shown that effective tracing contacts include in‐depth dialogue and navigation support [45, 46, 47]. Positive relationships between patients and healthcare workers and social support for continuity of care are also important for success [41, 44, 48, 49, 50]. Programmes may improve tracing effectiveness by identifying and targeting patients who are more susceptible to tracing and by understanding facility‐level influences that may mediate intervention effects [17, 18]. More data are needed on the mechanisms through which tracer–patient contact influences return.

To support re‐engagement, research is needed on appropriate combinations of tools [44, 51] and how to optimize support based on patient sub‐groups [52, 53] and time since LTFU. Targeting tracing efforts may help optimize their effectiveness. Targeting would be served by improving EMR data accuracy, including linking patient visits at different facilities to the same patient record. EMR accuracy would help identify truly disengaged patients by reclassifying those with undocumented facility transfers. Studies to identify indicators of likelihood to disengage [52], including reasons for loss [43, 54], could guide targeted tracing efforts and help maximize tracing effectiveness. A broad application of tracing may not warrant the required investment based on the limited causal effect. However, understanding which barriers tracing overcomes, and for whom, is critical to leveraging the benefit of tracing.

IV analysis validity depends on meeting IV assumptions. We empirically verified those assumptions that were verifiable. While unmeasured confounding, monotonicity and stable unit treatment value are ultimately unverifiable, they are highly plausible since we used randomization as an IV. Routine data sources present limitations, including missing data. We could not identify individuals who returned to a facility outside of the 71 BetterInfo study facilities or to one of our study sites under a newly assigned unique patient number. The EMR may not have captured some return visits due to missing data. Although this may underestimate return, misclassification would still be non‐differential by randomization status as outcome ascertainment were based solely on EHR data and not related to tracing activities. Approximately 30% of participants were ART naïve at loss. Tracing failed in approximately one‐third of patients randomized to tracing. Thus, the effect of tracer contact cannot be generalized to this group, which is likely to have worse HIV outcomes.

Per the BetterInfo tracing timeline, we could not examine the effect of tracer contact before 7 weeks post‐loss. Although findings were robust when restricted to an ART‐initiated sub‐group, the effect of tracer contact on return may not be generalizable in the treat‐all era. Similarly, to the extent that the effect of tracing is associated with the broader service delivery context, differences in HIV service delivery since 2017 limit the generalizability of study findings in programmes implementing revised practices. These differences include expanded differentiated care models, extended visit spacing, integration of HIV and other health services [55], and effects of COVID‐19 [56, 57, 58]. Research assessing tracing within current models is warranted to inform current practice.

## 5 CONCLUSIONS

As dynamic cycles of disengagement and re‐engagement are increasingly recognized as part of the HIV care experience [59, 60, 61], there is an urgent need for effective re‐engagement interventions. Our findings suggest that targeting tracing based on patient characteristics and trajectories may help optimize its effect. This likely requires improved EMR data and more sensitive assessments of out‐of‐care status. A better understanding of the mechanisms associated with return to care, intervention timing and targeting those likely to respond to tracing is needed to guide efficient health system practice.

## COMPETING INTERESTS

The authors declare no competing interests.

## AUTHORS’ CONTRIBUTIONS

LKB, EHG, CBM, IS and CM conceptualized and designed the study. The study procedures and data collection were implemented by KS, PS, SS and PK. Study analyses were completed by LKB with input from LHN, AM, SS, IEW, JP and JM. LKB wrote the manuscript. JAD, EHG, SS, IEW, AM, LHN and IS led review and substantive contributions to manuscript revisions, which all authors supported. All authors have read and approved the final manuscript.

## FUNDING

Research reported in this publication was supported by the National Institute of Mental Health of the National Institutes of Health under Award Number F31MH109378. This research was also supported by the Bill and Melinda Gates Foundation grant number OPP1105071 and the Johns Hopkins University Center for AIDS Research P30AI094189.

## DISCLAIMER

The content is solely the authors' responsibility and does not necessarily represent the official views of the National Institutes of Health.

## Supporting information


**Figure S1**. Population receiving treatment: tracer contact before return or two years of follow‐up

**Figure S2**. Instrumental Variable Estimate: Unadjustedˆ treatment effect of tracer contact on return to care among patients lost to follow‐up, risk difference, and 95% confidence interval by sampled health facility

**Figure S3**. Instrumental Variable Estimate: Adjustedˆ treatment effect of tracer contact on return to care among patients lost to follow‐up, risk difference, and 95% confidence interval by time from date of loss to randomization to tracing assignment

**Table S1**. Patient characteristics by randomization to tracing (instrumental variable) among patients lost as of the date of treatment randomization
**Table S2**. Treatment effect of tracer contact on return to care by two years post‐randomization among patients lost to follow‐up at date of treatment randomization
**Table S3**. Treatment effect of tracer contact on return to care by two years post‐loss among patients who had initiated ART before loss
**Table S4**. Instrumental Variable Estimate: Treatment effect of tracer contact on return to care among patients LTFU, full model estimates

## Data Availability

The Government of Zambia allows data sharing when applicable local conditions are satisfied. To request data access, contact the CIDRZ Ethics and Compliance Committee Chair/Chief Scientific Officer, Roma Chilengi (roma.chilengi@cidrz.org), or the Secretary to the Committee/Head of Research Operations, Hope Mwanyungwi (hope.mwanyungwi@cidrz.org), mentioning the intended use for the data.
