# Supplementary material for: The effect of tracer contact on return to care among adult, “lost to follow‐up” patients living with HIV in Zambia: an instrumental variable analysis
Source: J Int AIDS Soc. 2021 Dec 18;24(12):e25853. doi: 10.1002/jia2.25853 (PMC8683971; doi:10.1002/jia2.25853)
Supplement: Supplementary file 1 — Figure S1. Population receiving treatment: tracer contact before return or two years of follow‐up [file JIA2-24-e25853-s004.docx]

LTFU patients randomized to tracing:

4,380

250: Medical record review not completed

1,096: Ineligible for phone/household tracing, patient outcome ascertained from paper record review

*Outcome Disposition*

*In-care, original facility: 792*

*In-care, different facility: 186*

*Died: 118*

Paper medical record reviewed by tracer:

4,130

367: Phone / household tracing not possible, contact information unavailable

Eligible for phone / household tracing:

3,034

1,509: No patient-tracer contact prior to return or 2 years follow-up

*Outcome Disposition*

*Tracing failed: 675*

*Tracing determined patient died: 428*

*In-care, original facility*: 50*

*In-care, different facility*: 77*

*Alive, disengaged from care*: 88*

*Alive, unknown care status*: 191*

**Result determined by tracing contact that occurred after return or 2 years follow-up*

Phone / household tracing attempted:

2,667

Patient contacted by tracer prior to return or 2 years follow-up (treatment):

1,158

*Outcome Disposition*

*In-care, original facility: 155*

*In-care, different facility: 361*

*Alive, disengaged from care: 496*

*Alive, unknown care status: 146*
