# Supplementary figures and images for: The effect of tracer contact on return to care among adult, “lost to follow‐up” patients living with HIV in Zambia: an instrumental variable analysis
Source: J Int AIDS Soc. 2021 Dec 18;24(12):e25853. doi: 10.1002/jia2.25853 (PMC8683971; doi:10.1002/jia2.25853)

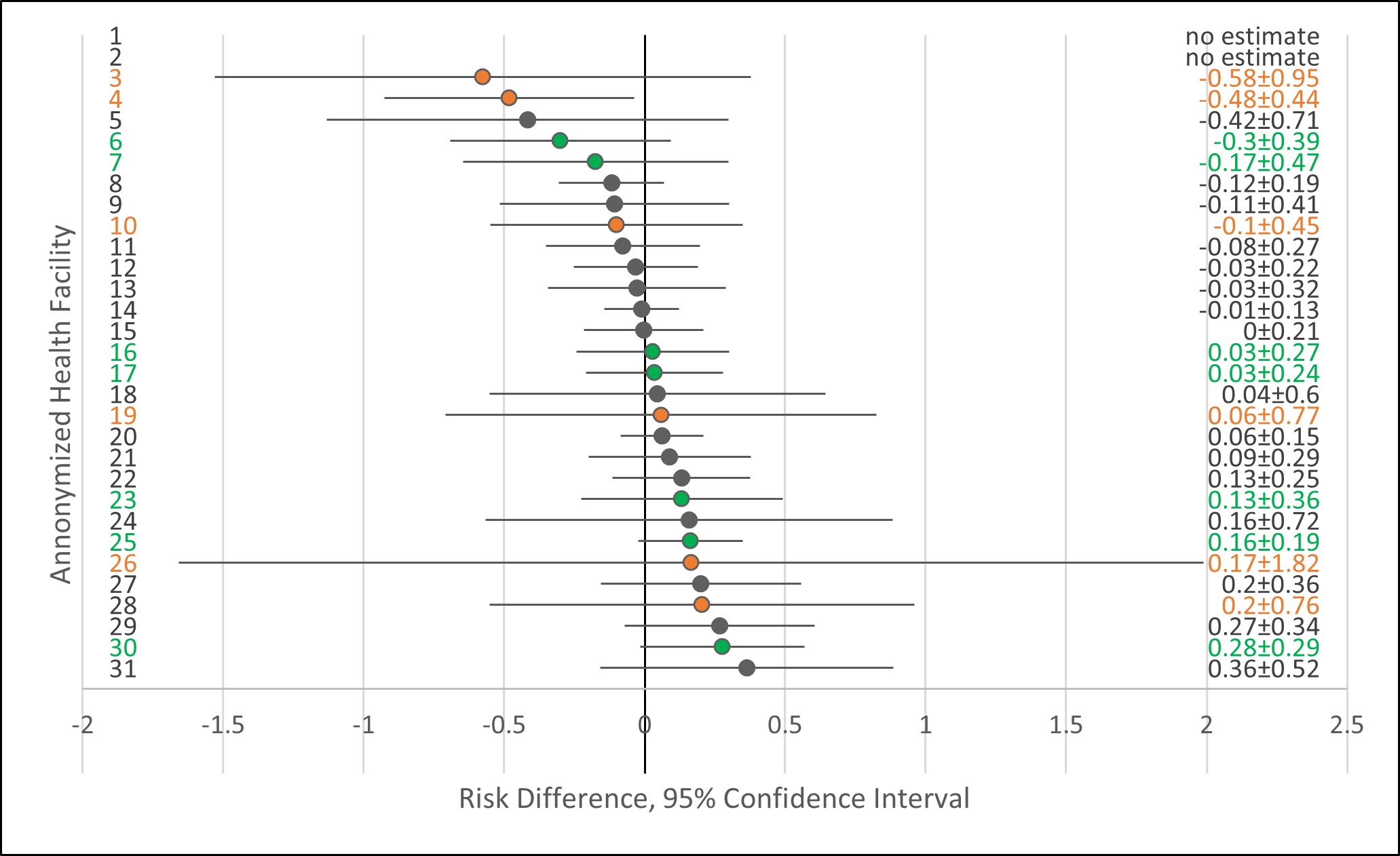

Supplement: Supplementary file 2 — Figure S2. Instrumental Variable Estimate: Unadjustedˆ treatment effect of tracer contact on return to care among patients lost to follow‐up, risk difference, and 95% confidence interval by sampled health facility [file JIA2-24-e25853-s002.png]

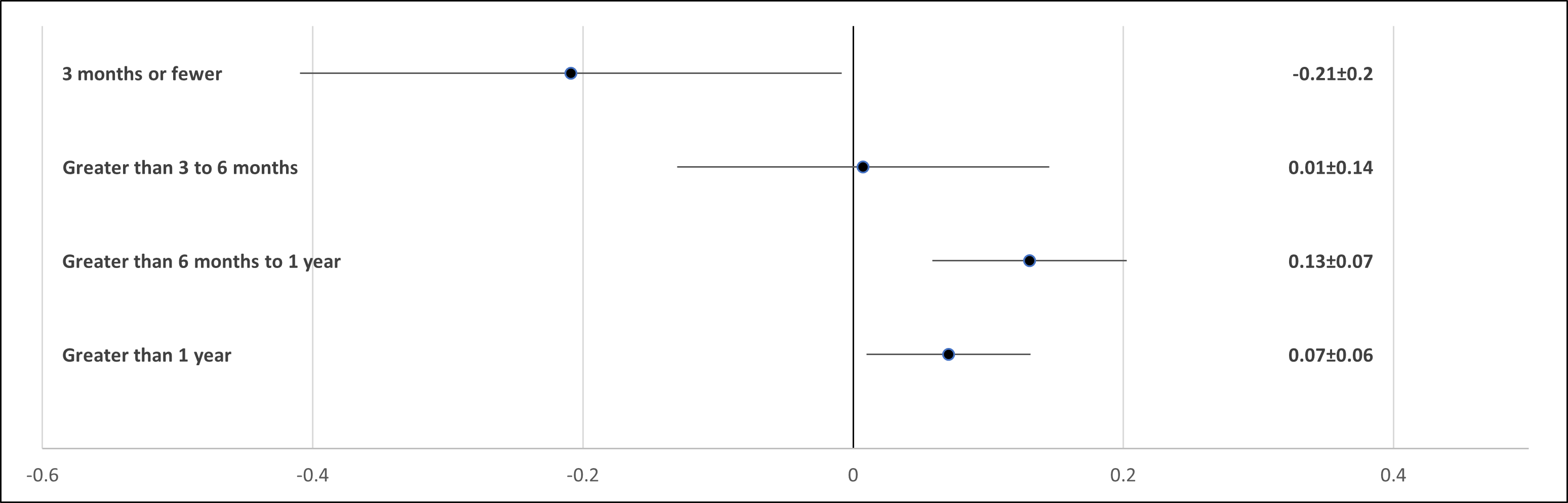

Supplement: Supplementary file 3 — Figure S3. Instrumental Variable Estimate: Adjustedˆ treatment effect of tracer contact on return to care among patients lost to follow‐up, risk difference, and 95% confidence interval by time from date of loss to randomization to tracing assignment [file JIA2-24-e25853-s001.png]
