# Supplementary material for: The effect of tracer contact on return to care among adult, “lost to follow‐up” patients living with HIV in Zambia: an instrumental variable analysis
Source: J Int AIDS Soc. 2021 Dec 18;24(12):e25853. doi: 10.1002/jia2.25853 (PMC8683971; doi:10.1002/jia2.25853)
Supplement: Supplementary file 4 — Table S1. Patient characteristics by randomization to tracing (instrumental variable) among patients lost as of the date of treatment randomization Table S2. Treatment effect of tracer contact on return to care by two years post‐randomization among patients lost to follow‐up at date of treatment randomization Table S3. Treatment effect of tracer contact on return to care by two years post‐loss among patients who had initiated ART before loss Table S4. Instrumental Variable Estimate: Treatment effect of tracer contact on return to care among patients LTFU, full model estimates [file JIA2-24-e25853-s003.docx]

**Supplemental Appendices**

**Supplemental Figure 1.**

**Supplemental Table 1. Patient characteristics by randomization to tracing (instrumental variable) among patients lost as of the date of treatment randomization^α^**

|  | **Not randomized to tracing** | | **Randomized to tracing** | | **Total** | **p-value*** |
| --- | --- | --- | --- | --- | --- | --- |
|  | **n=18,106** | | **n=4,009** | | **22,115** |  |
|  | **n** | **%** | **n** | **%** | **n** |  |
| *Characteristics* |  |  |  |  |  |  |
| **Sex** |  |  |  |  | 22,115 | 0.81 |
| Female | 10,842 | **60.1** | 2,409 | **60.1** | 13,251 |  |
| Male | 7,264 | **39.9** | 1,600 | **39.9** | 8,864 |  |
| **Age at loss (years)** |  |  |  |  | 22,055 | 0.09 |
| 18-24 | 1,947 | **10.8** | 450 | **11.3** | 2,397 |  |
| 25-34 | 6,923 | **38.3** | 1,469 | **36.8** | 8,392 |  |
| 35-44 | 6,038 | **33.4** | 1,317 | **33.0** | 7,355 |  |
| 45+ | 3,158 | **17.5** | 753 | **18.9** | 3,911 |  |
| **Ever initiated antiretroviral therapy** |  |  |  |  | 22,115 | <0.01 |
| No | 5,602 | **31.0** | 1,454 | **36.3** | 7,056 |  |
| Yes | 12,504 | **69.1** | 2,555 | **63.7** | 15,059 |  |
| **Weeks from time of enrollment to loss (mean, sd^1^)** | 17.5 | 18.1 | 15.9 | 16.7 | 22,115 | <0.01^ |
| **Number prior gaps in care** |  |  |  |  | 22,115 | <0.01 |
| 0 gaps | 9,756 | **53.9** | 2,335 | **58.2** | 12,091 |  |
| 1 gap | 4,597 | **25.4** | 959 | **23.9** | 5,556 |  |
| 2-3 gaps | 3,275 | **18.1** | 608 | **15.2** | 3,883 |  |
| 4 gaps or more | 478 | **2.6** | 107 | **2.7** | 585 |  |
| **Facility type** |  |  |  |  | 22,115 | <0.01 |
| Rural Health Center | 1,350 | **7.5** | 893 | **22.3** | 2,243 |  |
| Urban Health Center | 12,779 | **70.6** | 2,098 | **52.3** | 14,877 |  |
| Hospital | 3,977 | **22.0** | 1,018 | **25.4** | 4,995 |  |
| **Months from LTFU to randomization (mean, sd^1^)** | 10.2 | **5.9** | 10.2 | **5.9** | 22,115 | 0.82^ |
| **Marital status** |  |  |  |  | 18,049 | 0.64 |
| Single, Never Married | 2,006 | **13.6** | 473 | **14.4** |  |  |
| Married | 9,321 | **63.1** | 2,045 | **62.3** |  |  |
| Divorced | 2,153 | **14.9** | 477 | **14.5** |  |  |
| Widowed | 1,284 | **8.7** | 290 | **8.8** |  |  |
| **Education** |  |  |  |  | 17,518 | <0.01 |
| No formal education | 817 | **5.7** | 291 | **9.3** | 1,108 |  |
| Lower-mid Basic | 5,137 | **35.7** | 1,164 | **37.1** | 6,301 |  |
| Upper Basic - Secondary | 7,801 | **54.3** | 1,543 | **49.1** | 9,344 |  |
| Tertiary | 621 | **4.3** | 144 | **4.6** | 765 |  |
| **Last CD4 count (cells/µmol) before loss** |  |  |  |  | 18,742 | 0.09 |
| <=100 | 1,895 | **12.1** | 392 | **12.5** | 2,287 |  |
| 101-350 | 5,734 | **36.7** | 1,142 | **36.5** | 6,876 |  |
| 351-500 | 3,339 | **21.4** | 612 | **19.6** | 3,951 |  |
| >500 | 4,648 | **29.8** | 980 | **31.4** | 5,628 |  |
| *Treatment* |  |  |  |  |  |  |
| **Patient contacted by tracer before return or 2 years post-randomization** |  |  |  |  | 22,115 | <0.01 |
| No | 18,106 | **100.0** | 2,689 | **67.1** | 20,795 |  |
| Yes | 0 | **0.0** | 1320 | **32.9** | 1,320 |  |
| *Outcome* |  |  |  |  |  |  |
| **Return visit within 2 years of loss** |  |  |  |  | 22,115 | <0.01 |
| No | 14,083 | **77.8** | 3,017 | **75.3** | 17,100 |  |
| Yes | 4,023 | **22.2** | 992 | **24.7** | 5,015 |  |
| *chi-square test |  |  |  |  |  |  |
| ^t-test |  |  |  |  |  |  |

α Due to earlier follow-up database closure at one facility, 143 participants were 49 days short of the full two-year follow-up time from randomization in this sensitivity analysis. 1: standard deviation

**Supplemental Table 2. Treatment effect of tracer contact on return to care by two years post-randomization among patients lost to follow-up at date of treatment randomization**

|  | **Two-stage least squares regression, Adjusted*^** | | | | **Binary Probit regression, Adjusted*#** | | | | | |  |
| --- | --- | --- | --- | --- | --- | --- | --- | --- | --- | --- | --- |
|  | **Risk Difference** | **95% CI** | | **p-value** | **Risk Difference** | **95% CI** | | **p-value** | | |  |
| **Patient contacted by tracer before return or 2yrs follow-up v. not** | 0.04 | -0.002 | 0.08 | 0.07 | model failed to converge | | | | | |  |
| *adjusted for sex, age at LTFU, ART initiation status, time since HIV care enrollment, prior care gaps, facility type, highest education attained | | | | | | | | | | |  |
| First stage: adjusted risk difference: 0.33, 95%CI: 0.32-0.33 partial r2=0.28, F=8494.04, p=<0.01 | | | | | | |  | |  |  | |

^Ordinary least squares regression (OLS), Crude: 0.02 (95%CI: -0.01, 0.04, p=0.21); OLS, Adjusted*: 0.02 (95%CI:

-0.01, 0.04, p=0.16)

#Probit regression, Crude: 0.05 (95%CI: -0.03, 0.13, p=0.21); Probit regression, Adjusted: 0.07 (95%CI: 0.00, 0.15, p=0.07)

**Supplemental Table 3. Treatment effect of tracer contact on return to care by two years post-loss among patients who had initiated ART before loss, n=16,824**

|  | **Two-stage least squares regression, Adjusted*^** | | | | **Binary Probit regression, Adjusted*#** | | | |
| --- | --- | --- | --- | --- | --- | --- | --- | --- |
|  | **Risk Difference** | **95% CI** | | **p-value** | **Risk Difference** | **95% CI** | | **p-value** |
| **Patient contacted by tracer before return or 2 years follow-up v. not** | -0.01 | -0.10 | 0.07 | 0.77 | -0.01 | -0.10 | 0.07 | 0.76 |
| *adjusted for sex, age at LTFU, time since HIV care enrollment, prior care gaps, facility type, highest education attained, CD4 at last visit before loss | | | | | | | | |

First stage: adjusted risk difference: 0.22, 95%CI: 0.21-0.22 partial r2=0.18, F=3769.77, p=<0.01 ^Ordinary least squares regression (OLS), Crude: -0.09 (95%CI: -0.13, -0.05, p=<0.01); OLS, Adjusted*: -0.09 (95%CI: -0.13, -0.06, p=<0.01) #Probit regression, Crude: -0.27 (95%CI: -0.39, -0.16, p=<0.01); Probit regression, Adjusted: -0.28 (95%CI: -0.40, -0.17, p=<0.01)

**Supplemental Figure 2.** **Instrumental Variable Estimate: Unadjusted^^^ treatment effect of tracer contact on return to care among patients lost to follow-up, risk difference, and 95% confidence interval by sampled health facility***

**Supplemental Figure 3.** **Instrumental Variable Estimate: Adjusted^^^ treatment effect of tracer contact on return to care among patients lost to follow-up, risk difference, and 95% confidence interval by time from date of loss to randomization to tracing assignment***

**Supplemental Table 4. Instrumental Variable Estimate: Treatment effect of tracer contact on return to care among patients LTFU, full model estimates**

| **Characteristics** | **Risk Difference** | **95% CI** | | **p-value*** |
| --- | --- | --- | --- | --- |
| **Patient contacted by tracer before return or 2yrs follow-up v. none** | 0.03 | -0.02 | 0.08 | 0.23 |
| **Male Sex v. Female** | -0.03 | -0.04 | -0.02 | <0.01 |
| **Age at loss (years) v. 18-24 yrs** |  |  |  | <0.01 |
| 25-34 | 0.02 | 0.01 | 0.04 |  |
| 35-44 | 0.05 | 0.03 | 0.07 |  |
| 45+ | 0.05 | 0.03 | 0.07 |  |
| **Initiated ART before loss v. did not** | 0.06 | 0.05 | 0.07 | <0.01 |
| **Weeks from time of enrollment to loss (log)** | 0.06 | 0.05 | 0.07 | <0.01 |
| **Number prior gaps in care v. 0 gaps** |  |  |  | <0.01 |
| 1 gap | -0.01 | -0.02 | 0.01 |  |
| 2-3 gaps | 0.06 | 0.04 | 0.08 |  |
| 4 gaps or more | 0.18 | 0.15 | 0.22 |  |
| **Facility type v. rural health center** |  |  |  | <0.01 |
| Urban Health Center | -0.10 | -0.12 | -0.08 |  |
| Hospital | -0.09 | -0.11 | -0.07 |  |
| **Highest education attained v. none** |  |  |  | 0.09 |
| Lower-mid Basic | -0.01 | -0.04 | 0.01 |  |
| Upper Basic - Secondary | -0.01 | -0.04 | 0.01 |  |
| Tertiary | 0.02 | -0.02 | 0.05 |  |
| **Last CD4 count before loss v. <=100** |  |  |  | <0.01 |
| 101-350 | 0.07 | 0.05 | 0.08 |  |
| 351-500 | 0.11 | 0.09 | 0.13 |  |
| >500 | 0.15 | 0.13 | 0.17 |  |
